# Diagnostic accuracy of [^99*m*^Tc]pertechnetate scintigraphy in pediatric patients with suspected Meckel’s diverticulum: a 12-year, monocentric, retrospective experience

**DOI:** 10.3389/fmed.2025.1585313

**Published:** 2025-05-21

**Authors:** Bo Li, Jian Gao, Xintao Ding, Xiali Li, Yue Yu, Ye Long, Xiaohui Wang, Xinyu Wu, Yongju Gao

**Affiliations:** ^1^Department of Nuclear Medicine, Henan Key Laboratory of Novel Molecular Probes and Clinical Translation in Nuclear Medicine, Henan Provincial People’s Hospital, Zhengzhou University People’s Hospital, Henan University People’s Hospital, Zhengzhou, China; ^2^Department of Pediatric Surgery, Henan Provincial People’s Hospital, Zhengzhou University People’s Hospital, Henan University People’s Hospital, Zhengzhou, China; ^3^Department of Biomedical Informatics, Columbia University Graduate School of Arts and Sciences, New York, NY, United States; ^4^Business and Strategy Analytics, Progyny, Inc., New York, NY, United States

**Keywords:** [^99*m*^Tc]pertechnetate scintigraphy, Meckel’s scan, Meckel’s diverticulum, ectopic gastric mucosa, SPECT/CT

## Abstract

**Objective:**

To assess the diagnostic accuracy of [^99*m*^Tc]pertechnetate scintigraphy in pediatric patients with suspected Meckel’s diverticulum (MD).

**Methods:**

A retrospective study was conducted on 94 pediatric patients who presented with symptoms suggestive of MD and underwent [^99*m*^Tc]pertechnetate scintigraphy at Henan Provincial People’s Hospital between September 2012 and August 2024. For patients with high clinical suspicion and equivocal scintigraphy results, Single Photon Emission Computed Tomography/Computed Tomography (SPECT/CT) or repeat scintigraphy was conducted. Hemoglobin levels were measured in all patients, and their correlation with MD was analyzed. Sensitivity, specificity, positive predictive value (PPV), negative predictive value (NPV), and overall diagnostic accuracy were calculated by comparing scintigraphic results with surgical and histopathological findings.

**Results:**

Among the 94 patients, 20 (21.3%) had positive [^99*m*^Tc]pertechnetate scintigraphy, all confirmed as true positives through laparoscopic resection and histopathological examination. Of the 74 patients with negative scintigraphy results, 6 were found to be false negatives based on surgical findings, and 68 were confirmed as true negatives. Therefore, the sensitivity, specificity, PPV, and NPV were 76.9% (20/26), 100.0% (68/68), 100.0% (20/20), and 91.9% (68/74), respectively. The overall diagnostic accuracy was 93.6% (88/94). SPECT/CT was utilized in three cases, enhancing diagnostic precision in patients with equivocal planar imaging results. Repeat scintigraphy was performed in three patients with high clinical suspicion and negative initial scans, resulting in one additional positive diagnosis. MD patients exhibited significantly lower hemoglobin levels compared to non-MD patients (88.69 ± 20.39 g/L vs. 107.24 ± 29.28 g/L; *p* = 0.0009), with hemoglobin showing moderate predictive value (AUC = 0.70; 95% CI: 0.60–0.81).

**Conclusion:**

[^99*m*^Tc]pertechnetate scintigraphy is a highly specific and accurate tool for diagnosing MD in pediatric patients. However, due to the potential for false negatives, additional SPECT/CT imaging or repeat scintigraphy may be warranted in cases with high clinical suspicion.

## Introduction

Meckel’s diverticulum (MD) is a common congenital anomaly of the gastrointestinal tract, resulting from incomplete regression of the embryonic omphalomesenteric duct. It is located at the antimesenteric border of the ileum, typically within approximately 100 cm of the ileocecal valve (1). The prevalence of MD in the population is 2-3% (2). Most cases of MD are asymptomatic and are incidentally discovered during surgery. However, clinical symptoms occur in approximately 25-40% of cases, including bleeding, intestinal obstruction, diverticulitis, and perforation (3). Given the potential for serious complications, accurate, and timely diagnosis is crucial.

Diagnosis of symptomatic MD is often challenging, but [^99*m*^Tc]pertechnetate scintigraphy (also known as Meckel’s scan) can detect ectopic gastric mucosa. Approximately 90% of bleeding MD cases harbor ectopic mucosa, hence the utility of [^99*m*^Tc]pertechnetate scintigraphy as a non-invasive diagnostic modality crucial for MD since its introduction in 1970 (4). In cases of MD bleeding, typical findings on [^99*m*^Tc]pertechnetate scintigraphy include a focal area of abnormal radiotracer accumulation in the right lower abdomen, often concurrent with uptake in normal gastric mucosa and without change in appearance on sequential images (5). Small areas of ectopic gastric mucosa may be overlooked. Abnormal uptake may also be observed in other conditions such as duplications containing ectopic gastric mucosa, vascular malformations, intussusception, or inflammatory bowel disease. However, uptake in bleeding MD is typically more focal and intense. The literature reports wide sensitivity (60-100%) and specificity (22-100%) ranges for [^99*m*^Tc]pertechnetate scintigraphy in detecting MD, resulting in varied accuracy from 57 to 100% (6–25). Rosenthall et al. reported a false-negative rate of 50% in their study (26). These widely varying results have sparked debate regarding the reliability of [^99*m*^Tc]pertechnetate scintigraphy in ruling out bleeding MD.

Single Photon Emission Computed Tomography/Computed Tomography (SPECT/CT) enhances diagnostic accuracy by providing three-dimensional localization of radiotracer uptake, correlating functional data with anatomical structures, and reducing false positives/negatives caused by overlapping physiological activity or anatomical anomalies (27, 28). This approach is particularly valuable in cases with atypical findings on planar imaging or high clinical suspicion despite inconclusive results. Similarly, repeat scintigraphy is warranted when initial scans are negative but clinical symptoms, such as persistent gastrointestinal bleeding, suggest MD. Repeat imaging can mitigate false negatives caused by transient factors, such as rapid tracer washout during active bleeding or insufficient ectopic gastric mucosa activity during the initial scan (14, 29). These imaging approaches are critical for improving diagnostic confidence and guiding clinical management in pediatric patients with suspected MD.

We conducted a retrospective study at Henan Provincial People’s Hospital and Zhengzhou University People’s Hospital to evaluate the diagnostic accuracy of [^99*m*^Tc]pertechnetate scintigraphy in pediatric patients with suspected MD. Our study integrates clinical presentation, scintigraphic findings, histopathological confirmation, and the selective use of SPECT/CT and repeat scintigraphy to address equivocal or negative initial results, aiming to provide a comprehensive assessment of the utility of [^99*m*^Tc]pertechnetate scintigraphy.

## Patients and methods

### Patients

**FIGURE 1:**
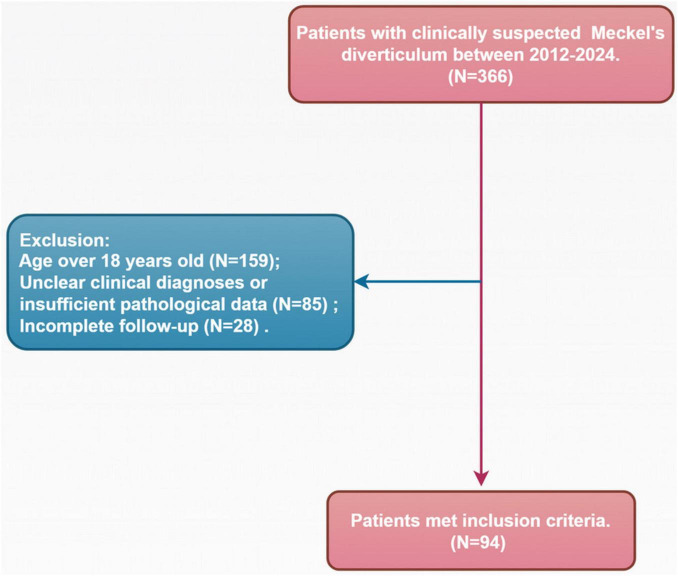
Flowchart of patient selection.

A retrospective study was conducted on 366 patients presenting with symptoms such as melena, abdominal pain, and other signs of lower gastrointestinal bleeding, suspected to be caused by MD. These patients underwent [^99*m*^Tc]pertechnetate scintigraphy in the Department of Nuclear Medicine at Henan Provincial People’s Hospital and Zhengzhou University People’s Hospital between September 2012 and August 2024. Data were retrieved through a comprehensive review of electronic medical records, including clinical notes, diagnostic imaging reports, and pathology results. Patients were excluded if they were over 18 years old (*n* = 159), had unclear clinical diagnoses or insufficient pathological data (*n* = 85), or had incomplete follow-up information (*n* = 28). Ultimately, 94 patients met the criteria for analysis. A detailed patient selection flowchart is presented in Figure 1. Table 1 summarizes the patients’ age at onset, gender, hemoglobin levels, primary symptoms (hematochezia, melena, abdominal pain, or severe anemia), final clinical diagnosis, and [^99*m*^Tc]pertechnetate scintigraphy results.

**TABLE 1:** Patient demographics and baseline characteristics.

Characteristic	*N* = 94^1^
**Age (year)**	11.5 (7.0, 13.8)
**Gender**	
Male	61 (64.9%)
Female	33 (35.1%)
**Hemoglobin (g/L)**	102 ± 28
**Primary symptom**	
Hematochezia	36 (38.3%)
Melena	18 (19.1%)
Abdominal pain	38 (40.4%)
Severe anemia	2 (2.1%)
**Final diagnosis**	
Meckel’s diverticulum	26 (27.7%)
Gastroenteritis	16 (17.0%)
Gastritis/duodenitis	14 (14.9%)
Colitis	6 (6.4%)
Necrotizing enterocolitis	1 (1.1%)
Incomplete intestinal obstruction	4 (4.3%)
Henoch-Schonlein purpura	3 (3.2%)
Juvenile polyps	2 (2.1%)
Mesenteric cysts	2 (2.1%)
Inflammatory bowel disease	2 (2.1%)
Cholangitis	1 (1.1%)
Cholecystitis	1 (1.1%)
Intra-abdominal infections	1 (1.1%)
Dieulafoy’s disease	1 (1.1%)
Hereditary Hemorrhagic Telangiectasia	1 (1.1%)
Nutcracker syndrome	1 (1.1%)
Gastricstromal tumor with digestive tract bleeding	1 (1.1%)
Small intestinal vascular malformations	1 (1.1%)
Unknown	10 (10.6%)
**[^99*m*^Tc]pertechnetate scintigraphy**	
Positive	20 (21.3%)
Negative	74 (78.7%)

^1^Median (IQR); Mean ± SD; n (%).

### [^99*m*^Tc]pertechnetate scintigraphy

Pediatric patients were instructed to fast for 3-6 h prior to scanning to reduce gastric volume. The use of perchlorate or laxatives was avoided before scintigraphy. No pharmacological interventions such as pentagastrin, histamine, glucagon, or H2-receptor antagonists were employed to enhance the results of [^99*m*^Tc]pertechnetate imaging in this study. After intravenous injection of 1.85 MBq/kg of [^99*m*^Tc]pertechnetate, anterior and posterior planar images of the abdomen and pelvis were obtained at 5, 10, 20, 30, 60, and 120 min post-injection using a low-energy high-resolution collimator (Discovery NM670, GE Healthcare, NY, United States). Images were acquired with a 128 × 128 matrix for 120 s per matrix, with age-appropriate zoom. Lateral images were obtained when necessary to assist in the localization of renal pelvis activity. A positive [^99*m*^Tc]pertechnetate scan was defined by abnormal focal tracer activity visible in early and delayed anterior images, not attributable to physiological activity, with intensity increasing over time in parallel with normal gastric mucosa activity. Repeat scintigraphy was performed on 3 patients with high clinical suspicion and initially negative scans. Additionally, 3 patients underwent abdominal and pelvic SPECT/CT scans. The fused images facilitated precise anatomical localization of uptake foci, preventing false positives due to urinary tract activity or vascular anomalies adjacent to bowel loops. In cases of negative scans, SPECT/CT could also identify alternative causes of abdominal pain and bleeding. SPECT imaging parameters were as follows: matrix size of 128 × 128, angular resolution of 6 degrees, and 30 s per step acquisition time. Low-dose CT scan parameters were 130 kV and 25 reference mAs, with images reconstructed using a B41s smooth kernel at a thickness of 5 mm. All [^99*m*^Tc]pertechnetate scintigraphies were independently interpreted by two experienced nuclear medicine physicians, with final diagnoses determined by consensus. Images were analyzed using MedEx software (MedEx Medical Ltd., Beijing, China).

### Statistical analysis

Data distribution was assessed using Shapiro-Wilk test and examination of histograms to confirm data skewness. Based on these assessments, appropriate descriptive statistics were applied for normally and non-normally distributed variables. Normally distributed continuous variables were presented as means and standard deviations (SD), while non-normally distributed continuous variables were presented as medians and interquartile ranges (IQR). Categorical variables were reported as counts and percentages. Continuous variables were analyzed using Student’s *t*-test or rank-sum test, and categorical variables were analyzed using the chi-square test. Based on histopathological results, the specificity, sensitivity, positive predictive value (PPV), negative predictive value (NPV), and overall diagnostic accuracy of [^99*m*^Tc]pertechnetate scintigraphy were calculated. The correlation between hemoglobin levels and MD was analyzed using logistic regression. Receiver operating characteristic (ROC) curve analysis was performed to evaluate the diagnostic performance of hemoglobin levels in predicting MD. A *p*-value of less than 0.05 was considered statistically significant. The violin plot was generated using the R package ggplot2. All statistical analyses were performed using R software (version 4.2.2).

## Results

### Patient characteristics and final diagnosis

Over a 12-year period, a total of 94 pediatric patients underwent [^99*m*^Tc]pertechnetate scintigraphy due to suspected MD. Of these patients, 61 (64.9%) were boys and 33 (35.1%) were girls, with a median age of 11.5 years (IQR: 7.0-13.8, range: 40 days to 17 years). Sixteen percent of the children were under 2 years old at the time of the scan, with 40% of them ultimately diagnosed with MD.

Among all patients who underwent [^99*m*^Tc]pertechnetate scans, hematochezia was the presenting symptom in 36 patients (38.3%), melena in 18 (19.1%), abdominal pain in 38 (40.4%), and severe anemia in 2 (2.1%). Of the 20 patients with positive scans (21.3%), 8 (40%) presented with hematochezia, while 12 (60%) had melena. In the 74 patients with negative scans, 28 (37.8%) presented with hematochezia, 6 (8.1%) with melena, 38 (51.4%) with abdominal pain, and 2 (2.7%) with severe anemia.

**FIGURE 2:**
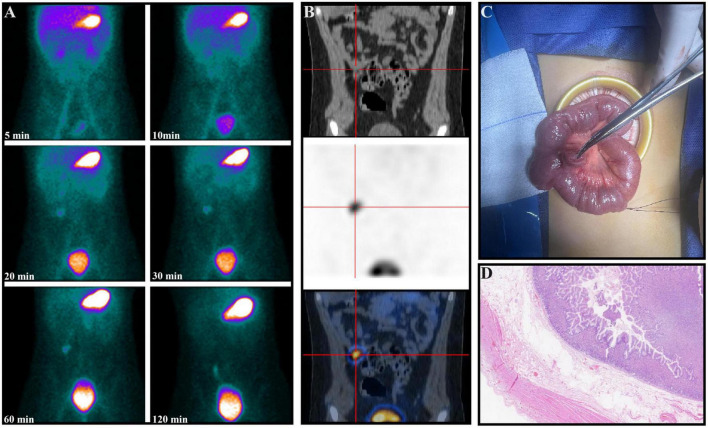
A 9-year-old boy presented with intermittent rectal bleeding and a hemoglobin level of 62 g/L. The serial images from [^99*m*^Tc]pertechnetate scintigraphy **(A)** show abnormal radiotracer uptake in the right lower quadrant, consistent with gastric uptake, which persisted on serial imaging at 120 min. Coronal SPECT/CT images localized the MD containing ectopic gastric mucosa **(B)**. An intraoperative photograph shows the diverticulum on the antimesenteric border of the ileum **(C)**. Postoperative pathology confirmed the presence of a diverticulum with ectopic gastric mucosa, accompanied by focal granulomatous tissue proliferation with patchy necrosis **(D)** (Hematoxylin and eosin, × 100).

All 20 (21.3%) patients with positive scintigraphy, all of whom were male, underwent laparoscopic examination. Each was found to have a MD, which was successfully resected (A representative case is shown in Figure 2). Histopathological analysis confirmed the presence of gastric mucosa in all cases. Among the 74 (78.7%) patients with negative scintigraphy, 6 underwent further endoscopic or laparoscopic evaluation, which identified MD in each case. Postoperative histology confirmed the presence of ectopic gastric mucosa, identifying these as false-negative results.

**FIGURE 3:**
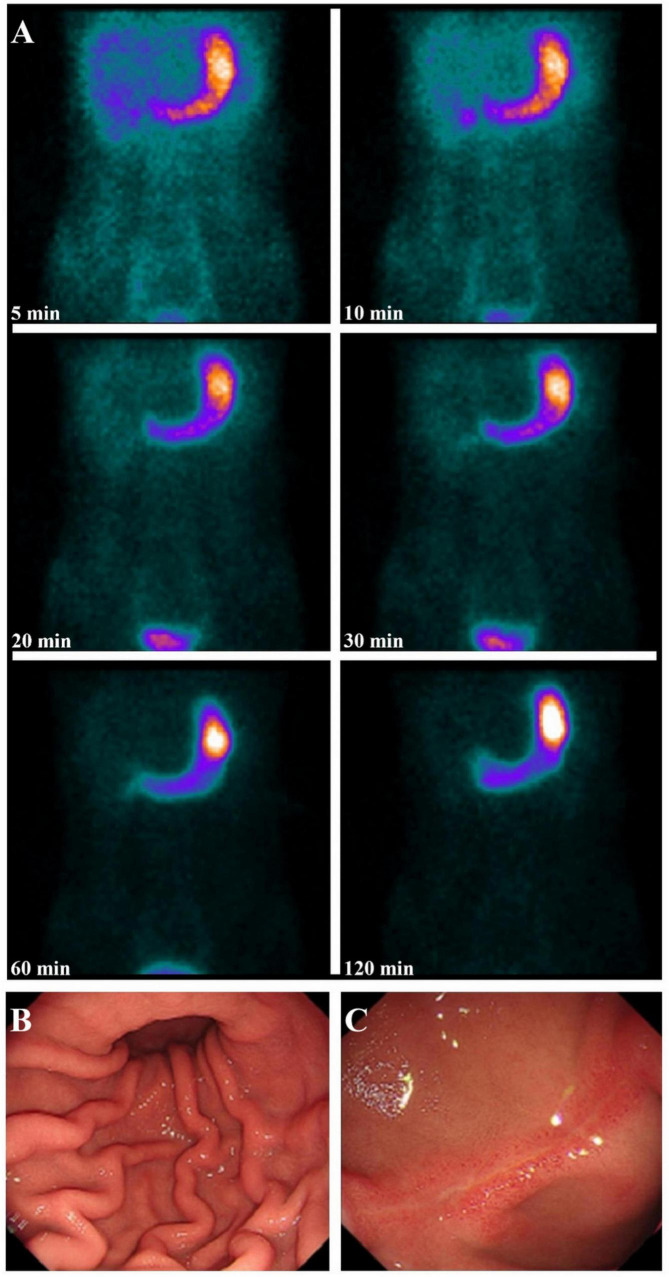
A 13-year-old boy presented with melena and a hemoglobin level of 150 g/L. Serial images from [^99*m*^Tc]pertechnetate scintigraphy showed no focal abnormal uptake suggestive of ectopic gastric mucosa in a MD **(A)**. Subsequent gastrointestinal endoscopy revealed scattered mucosal congestion in the gastric body **(B)** and a linear ulcer approximately 2 cm in length on the anterior wall of the duodenal bulb, with surrounding mucosal congestion and edema **(C)**. The patient was diagnosed with gastritis/duodenitis and improved following symptomatic treatment.

In 58 patients, alternative diagnoses were established following additional investigations, including imaging, endoscopy, and laparoscopy (A representative case is illustrated in Figure 3). These diagnoses included gastroenteritis (16 patients, 17.0%), gastritis/duodenitis (14 patients, 14.9%), colitis (6 patients, 6.4%), necrotizing enterocolitis (1 patient, 1.1%), incomplete intestinal obstruction (4 patients, 4.3%), Henoch-Schonlein purpura (3 patients, 3.2%), juvenile polyps (2 patients, 2.1%), mesenteric cysts (2 patients, 2.1%), inflammatory bowel disease (2 patients, 2.1%), cholangitis (1 patient, 1.1%), cholecystitis (1 patient, 1.1%), intra-abdominal infection (1 patient, 1.1%), Dieulafoy’s disease (1 patient, 1.1%), hereditary hemorrhagic telangiectasia (1 patient, 1.1%), nutcracker syndrome (1 patient, 1.1%), gastrointestinal stromal tumor with digestive tract bleeding (1 patient, 1.1%) and small intestinal vascular malformation (1 patient, 1.1%) (Table 1).

For the remaining 10 patients, despite extensive medical evaluation, no definitive diagnosis was established. However, all symptoms resolved without recurrence during follow-up, which ranged from 8 months to 3 years. In this group, the likelihood of MD-related bleeding appears to be low.

### Diagnostic accuracy of [^99*m*^Tc]pertechnetate scintigraphy

All 20 positive [^99*m*^Tc]pertechnetate scintigraphies results were confirmed to be true positive at pathological examination. Among the 74 negative scintigraphies, 6 were identified as false negative based on postoperative pathology, while the remaining 68 were confirmed as true negative through clinical evaluation, imaging studies, endoscopic, and laparoscopic assessments.

Therefore, the sensitivity, specificity, PPV, and NPV of [^99*m*^Tc]pertechnetate scintigraphy for diagnosing MD were 77.0% (20/26), 100.0% (68/68), 100.0% (20/20), and 91.9% (68/74), respectively. The overall diagnostic accuracy was 93.6% (88/94) (Table 2).

**TABLE 2:** The diagnosis accuracy of [^99*m*^Tc]pertechnetate scintigraphy in the diagnosis of Meckel’s diverticulum.

[^99*m*^Tc] pertechnetate scintigraphy	Final diagnosis		Index
	**MD, n (%)**	**Non-MD, n (%)**	
Positive, n (%)	20 (21.3)	0 (0.0)	PPV = 100.0%
Negative, n (%)	6 (6.4)	68 (72.3)	NPV = 91.9%
Index	Se = 77.0%	Sp = 100.0%	Ac = 93.6%

Se, sensitivity; Sp, specificity; PPV, positive predictive value; NPV, negative predictive value; Ac, accuracy.

### SPECT/CT and repeat imaging

In one pediatric patient with severe hematochezia, SPECT/CT successfully identified and precisely localized the [^99*m*^Tc]pertechnetate scintigraphy-positive lesion (Figure 2). Additionally, in two patients with negative initial scintigraphy results, SPECT/CT provided further diagnostic clarity: one case of abdominal pain with melena was subsequently diagnosed as gastrointestinal stromal tumor with hemorrhage, and another case presenting with abdominal pain was ultimately attributed to peritonitis secondary to intra-abdominal infection.

**FIGURE 4:**
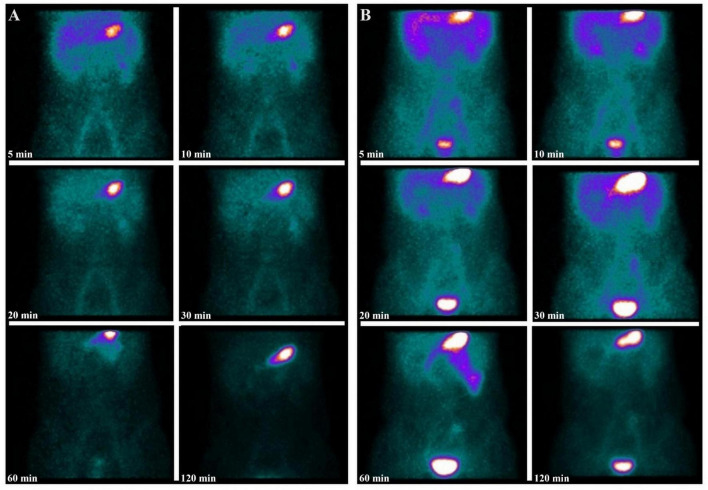
A 12-year-old boy presented with intermittent rectal bleeding and a hemoglobin level of 117 g/L. The initial serial images from [^99*m*^Tc]pertechnetate scintigraphy showed physiological radiotracer activity in the liver, stomach, and bladder, with no focal abnormal uptake suggestive of ectopic gastric mucosa in a MD **(A)**. Given the lack of symptomatic improvement with supportive clinical management, a high suspicion of MD persisted. A repeat [^99*m*^Tc]pertechnetate scintigraphy was performed, revealing a relatively fixed area of increased radiotracer uptake in the left lower quadrant **(B)**. The diagnosis of MD was confirmed postoperatively. Upon further inquiry, the patient’s family revealed that the patient had experienced significant rectal bleeding prior to the initial [^99*m*^Tc]pertechnetate scintigraphy, suggesting that the negative initial results might have been due to dilution of the secreted [^99*m*^Tc]pertechnetate by the bleeding.

Among three patients who initially presented with negative scintigraphy but were strongly suspected of having MD, repeat [^99*m*^Tc]pertechnetate scintigraphy was performed. One patient exhibited a positive result upon repeat scanning (Figure 4), potentially due to the rapid washout of [^99*m*^Tc]pertechnetate during an active bleeding episode in the initial scan, which may have led to a false negative result (3). The remaining two patients continued to exhibit negative results on repeat scanning. Subsequent endoscopic evaluation diagnosed one with inflammatory bowel disease, while the other patient experienced symptom resolution with supportive care and demonstrated no further episodes of abdominal pain or hematochezia on follow-up, effectively excluding MD.

### Hemoglobin levels

**FIGURE 5:**
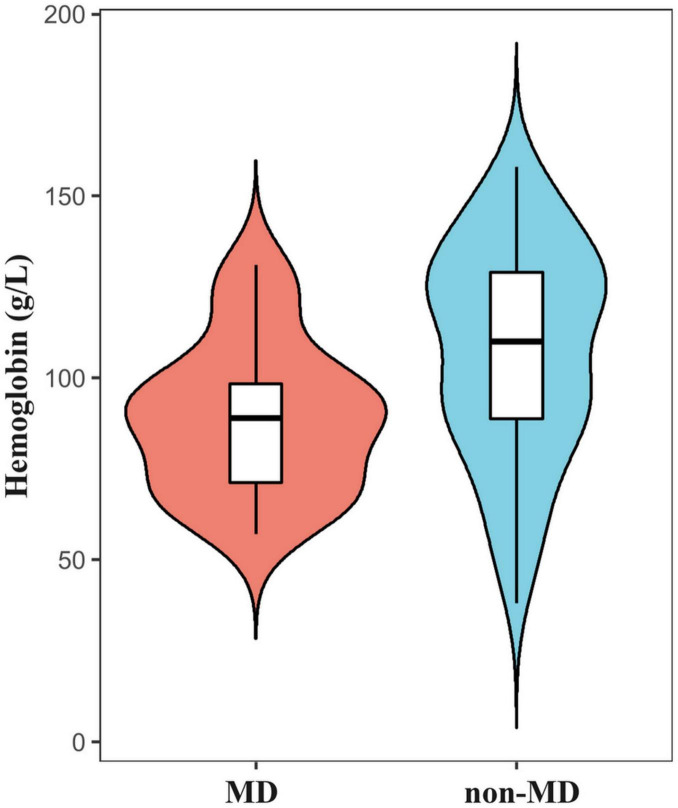
Violin plot illustrating that the mean hemoglobin level in patients diagnosed with MD was significantly lower than that in non-MD patients.

**FIGURE 6:**
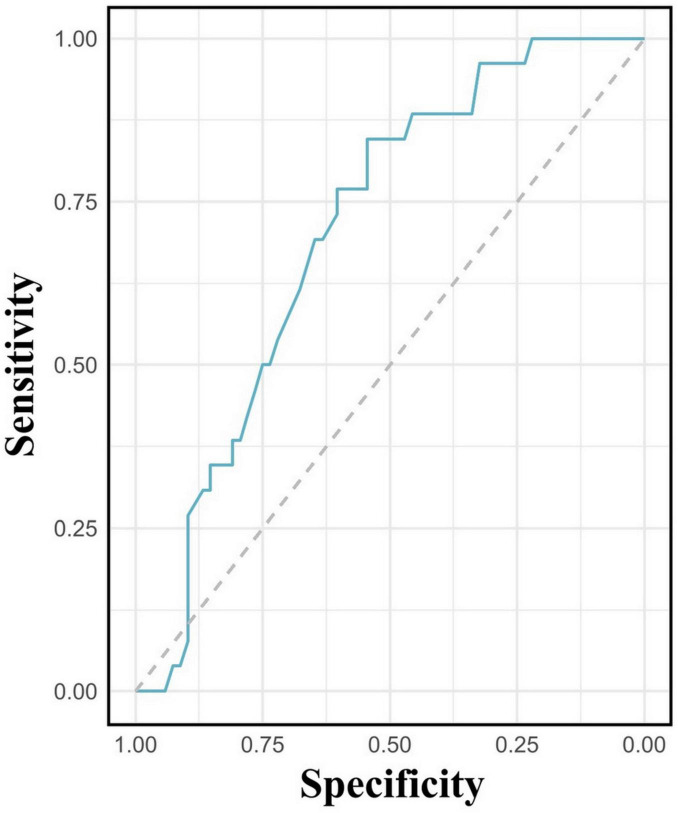
Receiver operating characteristic curve evaluating hemoglobin levels in predicting Meckel’s diverticulum.

In this study, 55 patients (58.5%) presented with hemoglobin levels below the normal range. The mean hemoglobin level among patients diagnosed with MD was 88.69 ± 20.39 g/L, significantly lower than the mean level of 107.24 ± 29.28 g/L observed in non-MD patients (*p* = 0.0009) (Figure 5). Lower hemoglobin levels were significantly associated with MD (OR = 0.98, 95% CI: 0.96-0.99; *p* = 0.006). ROC analysis demonstrated moderate utility of hemoglobin levels in predicting MD (AUC = 0.70, (95% CI: 0.60-0.81) (Figure 6).

## Discussion

Our findings demonstrate a high overall diagnostic accuracy of 93.6% for [^99*m*^Tc]pertechnetate scintigraphy in pediatric patients with suspected MD. These results are consistent with previously reported data, where sensitivity ranges from 60 to 100% and specificity from 22 to 100% (6–25) (as summarized in Table 3). The specificity of 100.0% in our cohort underscores the reliability of [^99*m*^Tc]pertechnetate scintigraphy in confirming MD when the scan is positive, as evidenced by a PPV of 100.0%. However, a sensitivity of 77.0% and a NPV of 91.9% indicate that a negative scan cannot definitively rule out MD, particularly in symptomatic patients with ongoing clinical suspicion. The presence of six false negative cases in our study highlights the need for caution when interpreting negative scintigraphy results.

**TABLE 3:** Summary detection accuracy of series reporting [^99*m*^Tc]pertechnetate scintigraphy in the diagnosis of Meckel’s diverticulum.

First author, country, year of publication	Study design	*N*	Se	Sp	PPV	NPV	Ac	Reference
Leonidas J C, United States, 1974	Retrospective	13	75.0% (3/4)	100% (9/9)	100% (3/3)	90.0% (9/10)	92.3% (12/13)	(6)
Ho JE, United States, 1975	Retrospective	20	100% (3/3)	100% (17/17)	100% (3/3)	100.0% (17/17)	100.0% (20/20)	(7)
Gelfand M J, United States, 1978	Retrospective	55	77.8% (7/9)	95.7% (44/46)	77.8% (7/9)	95.7% (44/46)	92.7% (51/55)	(8)
Cooney D R, United States, 1982	Retrospective	266	85.7% (12/14)	94.4% (238/252)	46.2% (12/26)	99.2% (238/240)	94.0% (250/266)	(9)
Fries M, SWEDEN, 1984	Retrospective	22	75.0% (9/12)	100% (10/10)	100% (9/9)	76.9% (10/13)	86.4% (19/22)	(10)
Kong MS, Taiwan (China), 1994	Retrospective	101	83.0% (39/47)	96.3% (52/54)	95.1% (39/41)	86.7% (52/60)	90.1% (91/101)	(11)
McCulley S, South Africa, 1996	Retrospective	77	66.7% (4/6)	91.5% (65/71)	40.0% (4/10)	97.0% (65/67)	89.6% (69/77)	(12)
Rampin L, Italian, 1998	NR	28	100% (10/10)	100% (18/18)	100% (10/10)	100% (18/18)	100% (28/28)	(13)
Swaniker F, United States, 1999	Retrospective	43	60.0% (12/20)	100% (23/23)	100% (12/12)	74.2% (23/31)	81.4% (35/43)	(14)
Poulsen KA, Denmark, 2000	Retrospective	55	60.0% (3/5)	98.0% (49/50)	75.% (3/4)	96.1% (49/51)	94.5% (52/55)	(15)
Rerksuppaphol S, Australia, 2004	Retrospective	9	77.8% (7/9)	100% (0/0)	100% (7/7)	0.0% (0/2)	77.8% (7/9)	(16)
Shalaby RY, Egypt, 2005	Retrospective	7	60.0% (3/5)	50.0% (1/2)	75.% (3/4)	33.3% (1/3)	57.1% (4/7)	(17)
Dolezal J, Czech Republic, 2008	Retrospective	79	100% (3/3)	100% (76/76)	100% (3/3)	100% (76/76)	100.0% (79/79)	(18)
Sinha CK, United Kingdom, 2013	Retrospective	183	94.4% (17/18)	97.0% (160/165)	77.3% (17/22)	99.4% (160/161)	96.7% (177/183)	(19)
Rho JH, Korea, 2013	Retrospective	12	91.7% (11/12)	100% (0/0)	100% (11/11)	0.0% (0/1)	91.7% (11/12)	(20)
Al Janabi M, United Kingdom, 2014	Prospective	61	84.2% (32/38)	21.7% (5/23)	64.0% (32/50)	45.5% (5/11)	60.7% (37/61)	(21)
Papparella A, Italy, 2014	Retrospective	19	64.7% (11/17)	50.0% (1/2)	91.7% (11/12)	14.3% (1/7)	63.2% (12/19)	(22)
Sancar, Turkey, 2015	Retrospective	8	80.0% (4/5)	33.3% (1/3)	66.7% (4/6)	50.0% (1/2)	62.5% (5/8)	(23)
Irvine I, Ireland, 2017	Retrospective	144	100% (22/22)	100% (122/122)	100% (22/22)	100% (122/122)	100.0% (144/144)	(24)
Chen Q, China, 2018	Retrospective	78	70.5% (55/78)	100% (0/0)	100% (55/55)	0.0% (0/23)	70.5% (55/78)	(25)
Li B, China, 2025	Retrospective	94	76.9% (20/26)	100% (68/68)	100% (20/20)	91.9% (68/74)	93.6% (88/94)	Present study

N, number of patients; NR, not reported; Se, sensitivity; Sp, specificity; PPV, positive predictive value; NPV, negative predictive value; Ac, accuracy.

**FIGURE 7:**
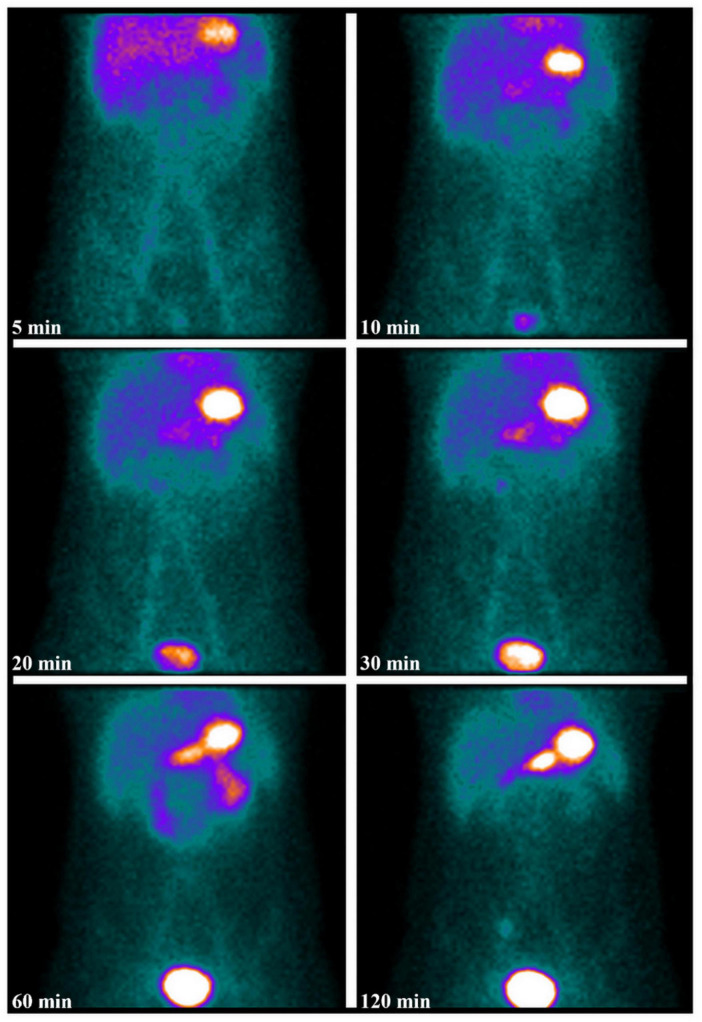
A 10-year-old boy presented with intermittent rectal bleeding and melena, with a hemoglobin level of 71 g/L. Serial [^99*m*^Tc]pertechnetate scintigraphy images obtained within 60 min showed no significant abnormal radiotracer accumulation in the abdomen or pelvis; however, delayed imaging at 120 min revealed abnormal radiotracer uptake in the right lower quadrant. The diagnosis of MD was confirmed postoperatively. A review of the patient’s history revealed that he had undergone gastrointestinal bleeding scintigraphy (*in vivo* red blood cell labeling scan) at an outside hospital 1 day prior, which may have influenced the results of the [^99*m*^Tc]pertechnetate scintigraphy.

The accuracy of Meckel’s scan largely depends on the presence of ectopic gastric mucosa in the MD, which selectively takes up the radiotracer and secretes it into the lumen of the diverticulum. For the gamma camera to detect and capture this activity, the ectopic gastric mucosa must not only be functional but also of sufficient size, approximately 1.8 cm^2^, to be identifiable (30, 31). The sensitivity of [^99*m*^Tc]pertechnetate scintigraphy is therefore, contingent on both the quantity and functionality of the ectopic gastric mucosa in the MD. Previous studies have indicated that false negatives may be associated with factors such as impaired radiotracer uptake due to poor function or low concentration of ectopic gastric mucosa, high gastric background causing decreased visibility of ectopic tissue, dilution of the secreted isotope due to bleeding (as observed in our study, Figure 4), and rapid transit caused by peristalsis or inflammation (32). Datz et al. have suggested that poor function of ectopic gastric mucosa may result from autonecrosis caused by its own secretion of hydrochloric acid and pepsin or from mucosal ulceration induced by enteroliths, which can lead to bleeding and impair tracer uptake (33). In our study, the patient depicted in Figure 2 may exemplify this phenomenon, as the necrosis observed at pathology could explain the relatively modest tracer accumulation. Furthermore, previous studies have suggested that the use of labeled red blood cell scans for detecting gastrointestinal bleeding may interfere with the accuracy of [^99*m*^Tc]pertechnetate scans. This is partly due to the possibility that [^99*m*^Tc]pertechnetate might label red blood cells instead of targeting ectopic gastric mucosa (34). The late visualization of the MD in one of our patients (as shown in Figure 7), after he underwent gastrointestinal bleeding scintigraphy 1 day before the scan, may relate to this effect. Similar challenges have been reported in the literature, with studies by Dolezal et al. (34) and Aboughalia et al. (35) emphasizing the importance of clinical judgment and the potential need for additional diagnostic tools in cases with negative scans but ongoing suspicion of MD.

In our study, SPECT/CT was performed in only 3 out of 94 patients, correlating positive planar findings with anatomical structures, and identifying alternative diagnoses in two true-negative cases. SPECT/CT may reduce the likelihood of false positives/negatives and thus increase overall diagnostic confidence. Dillman et al. (27) and Stathaki et al. (28) reported that SPECT/CT improves the detection of MD, particularly in cases with equivocal or negative planar imaging results. The utility of SPECT/CT in our cohort underscores its value as an adjunctive imaging modality, especially in complex cases where conventional scintigraphy is inconclusive. In such situations, it is crucial to tailor SPECT/CT protocols to minimize radiation exposure, particularly in vulnerable pediatric populations (27). Typically, sedation is not required for Meckel scans, and infant patients can be swaddled or placed in an infant bag to prevent excessive movement. However, the extended imaging time required for SPECT/CT may necessitate sedation, with its inherent risks. Additionally, the risk of mismatch between SPECT and CT images due to intestinal loop movement during acquisition can affect localization accuracy. Therefore, judicious use of SPECT/CT, tailored to the individual case, is an important technical consideration (36).

Our study highlights the potential benefit of repeat [^99*m*^Tc]pertechnetate scintigraphy in cases with persistent clinical suspicion of MD despite an initial negative imaging result. In one instance, a repeat scan led to a positive diagnosis after the initial scan failed to detect the diverticulum, likely due to the timing of the scan in relation to the bleeding episode. This observation is consistent with findings from Swaniker et al. (14), who suggested that repeated imaging could be crucial in patients with ongoing symptoms. Vali et al. (29) retrospectively evaluated 33 cases that underwent a second Meckel scan due to either equivocal results (12 cases) or persistent high clinical suspicion (21 cases). Of these, 9 cases had positive findings on repeat imaging, underscoring the utility of a second scintigraphy study. Therefore, repeat scintigraphy or alternative imaging modalities should be considered when clinical suspicion remains high.

Among the 20 patients with positive scans, 8 (40%) presented with hematochezia, and 12 (60%) with melena. Of the 6 false-negative cases, 5 had hematochezia, while 1 presented with both melena and hematochezia. Anemia was present in 85.0% (22/26) of patients with MD, with an average hemoglobin level of 89 ± 20 g/L, significantly lower than in non-MD patients (*p* = 0.0009). Although a substantial proportion of true-negative patients also experienced hematochezia, the occurrence of overt bleeding was less common in this group. Additionally, our study demonstrates a significant association between lower hemoglobin levels and the presence of MD, suggesting that anemia may serve as an additional diagnostic indicator. The notably lower average hemoglobin levels in patients with MD suggest that, in order to enhance sensitivity, the presence of anemia should prompt further diagnostic investigations, such as SPECT/CT or repeat scintigraphy, in cases with negative initial scintigraphy.

Some misleading comments from the surgical literature suggest that the negative predictive value of Meckel’s scan is poor and that it should not be relied upon to exclude MD. However, upon closer examination of these studies, we found that either their methodology for evaluating sensitivity was flawed, or they did not adhere to current international guidelines when conducting their scans (21, 37). Interpretation of [^99*m*^Tc]pertechnetate scintigraphy can be influenced by several factors, including patient preparation, the presence of interfering conditions, and the experience of the interpreting physician (35).

This study has several limitations, including its retrospective design and the fact that it was conducted at a single center, which may limit the generalizability of our findings. Additionally, the relatively small sample size, particularly in false negative cases, may limit the statistical power of our conclusions. Furthermore, several studies suggest that certain medications, such as H2 antagonists, 5-glutathione, and glucagon, can enhance the detection of ectopic gastric mucosa, potentially influencing diagnostic accuracy. However, our retrospective study did not include these interventions. Future prospective studies are needed to validate these findings and further refine diagnostic protocols.

## Conclusion

In conclusion, [^99*m*^Tc]pertechnetate scintigraphy remains a highly specific and effective tool for diagnosing MD. However, its limitations, particularly in sensitivity, necessitate the use of adjunctive imaging techniques such as SPECT/CT and the consideration of repeat scanning in certain clinical scenarios. These findings highlight the need for a multifaceted diagnostic approach and optimizing imaging protocols to ensure timely and accurate diagnosis, ultimately improving patient outcomes.

## Data Availability

The original contributions presented in this study are included in this article/supplementary material, further inquiries can be directed to the corresponding authors.
